# A phenomenological model of non-genomic variability of luminescent bacterial cells

**DOI:** 10.18699/VJGB-23-102

**Published:** 2023-12

**Authors:** S.I. Bartsev

**Affiliations:** Institute of Biophysics of the Siberian Branch of the Russian Academy of Sciences, Federal Research Center “Krasnoyarsk Science Center SB RAS”, Krasnoyarsk, Russia Siberian Federal University, Krasnoyarsk, Russia

**Keywords:** non-genomic variability, phenomenological model, luminescent bacteria, негеномная изменчивость, феноменологическая модель, люминесцентные бактерии

## Abstract

The light emitted by a luminescent bacterium serves as a unique native channel of information regarding the intracellular processes within the individual cell. In the presence of highly sensitive equipment, it is possible to obtain the distribution of bacterial culture cells by the intensity of light emission, which correlates with the amount of luciferase in the cells. When growing on rich media, the luminescence intensity of individual cells of brightly luminous strains of the luminescent bacteria Photobacterium leiognathi and Ph. phosporeum reaches 104–105 quanta/s. The signal of such intensity can be registered using sensitive photometric equipment. All experiments were carried out with bacterial clones (genetically homogeneous populations). A typical dynamics of luminous bacterial cells distributions with respect to intensity of light emission at various stages of batch culture growth in a liquid medium was obtained. To describe experimental distributions, a phenomenological model that links the light of a bacterial cell with the history of events at the molecular level was constructed. The proposed phenomenological model with a minimum number of fitting parameters (1.5) provides a satisfactory description of the complex process of formation of cell distributions by luminescence intensity at different stages of bacterial culture growth. This may be an indication that the structure of the model describes some essential processes of the real system. Since in the process of division all cells go through the stage of release of all regulatory molecules from the DNA molecule, the resulting distributions can be attributed not only to luciferase, but also to other proteins of constitutive (and not only) synthesis.

## Abstract (ru)

Свет, испускаемый люминесцентными бактериями, может служить уникальным природным каналом передачи информации о процессах внутри отдельной клетки. При наличии высокочувствительного оборудования можно получить распределение клеток бактериальной культуры по интенсивности свечения, которая коррелирует с количеством люциферазы в клетках. При выращивании на богатых питательных средах интенсивность свечения отдельных клеток ярко светящихся штаммов люминесцентных бактерий Photobacterium leiognathi и Ph. phosporeum достигает 104–105 квантов/с. Сигнал такой интенсивности может быть зарегистрирован с помощью чувствительного фотометрического оборудования. Все эксперименты проводились с бактериальными клонами – генетически однородными популяциями. Получена типичная динамика распределения светящихся бактериальных клеток по интенсивности свечения на различных стадиях периодического выращивания культуры в жидкой среде. Для описания экспериментальных распределений была построена феноменологическая модель, которая связывает излучение бактериальной клетки с историей событий на молекулярном уровне. Предложенная феноменологическая модель с минимальным числом подстроечных параметров (1.5) обеспечивает удовлетворительное описание сложного процесса формирования распределения клеток по интенсивности свечения на разных стадиях роста бактериальной культуры. Это может свидетельствовать о том, что структура модели описывает некоторые существенные процессы реальной системы. Поскольку в процессе деления все клетки проходят стадию отсоединения всех регуляторных молекул от молекулы ДНК, результирующие распределения можно отнести не только к люциферазе, но и к другим белкам конститутивного (и не только) синтеза.

## Introduction

The heterogeneity of isogenic bacterial populations, or, in other words, non-genomic variability of cells, is increasingly attracting the attention of researchers. This is partly due to the development of methods for tracking individual cell parameters, down to the dynamics of protein synthesis during the cell cycle (Taheri-Araghi et al., 2015; Andryukov et al., 2021). On the other hand, understanding the mechanisms or causes of phenotypic differences of cells from an isogenic population is important both for the formation of fundamental concepts of intracellular processes organization and for increasing the efficiency of solving practical problems in medicine and biotechnology.

The cell cycle is a potentially significant source of nongenomic variability. During the cell cycle, the protein abundance in the cell undergoes two-fold changes. In the case of an asynchronous population, these changes can contribute significantly to phenotypic variability. However, another possible source of heterogeneity is related to the cell cycle. It has been shown quite a long time ago (Shkolnik, 1989) that the widely used allometric dependences (when different variables Ni are related by relations of the form Ni = αiNβi 1 ), when describing growth curves, lead to a contradiction with observations. So in the case of an allometric growth model, a cell dies after a small number of generations due to the fact that certain substances abundance approaches zero. Then a phenomenological trigger model combining allometric growth with switches was proposed. According to the model, the passage of a cell through various phases of the cell cycle is accompanied by sharp changes in the allometric ratios of growth variables. There are certain combinations of parameters that can be conditionally associated with multidimensional switching surfaces – the boundaries of cellular phases – from cell birth to division. When passing the next boundary, the rates of change in cellular variables switch. This model was further developed (Zinovyev et al., 2022) and demonstrated strong agreement with experimental data.

According to this model, switching should occur in a certain sequence and in a fairly uniform manner, but for a nonsynchronous culture such switching can make a significant contribution to the variability of phenotypic traits. However, it should be noted that this model was compared with data on the dynamics of variable eukaryotic cells and it is possible that in bacterial cells the limitations of allometric growth are overcome in another way.

Thus, experimental observations of protein synthesis inside bacterial cells (Kiviet et al., 2014) show that the activation of particular protein synthesis occurs without pronounced patterns. Another paper on the topic (Walker et al., 2016) notes that the contribution of the bacterial cell cycle to expression noise consists of two parts: a deterministic fluctuation synchronous with the cell cycle and a stochastic component caused by variable timing of gene replication. It was shown earlier (Taniguchi et al., 2010) that proteins with strong expression have a coefficient of variation of ~30 %, which indicates an “external” factor not associated with fluctuations in the abundance of a small number of molecules.

Fluorescence microscopy is primarily used to monitor protein synthesis at the single-cell scale, which is essential for studying non-genomic variation. However, it is noted that with the current level of device sensitivity stimulating light has a negative effect on the physiological state of cells (Taheri- Araghi et al., 2015).

A unique alternative to fluorescence microscopy is the use of luminescence of luminescent bacteria (Deryabin, 2009) as a channel of information about the state of intracellular processes (Berzhanskaya et al., 1975; Bartsev, Gitelzon, 1985). The uniqueness of luminescence lies in the fact that the cell emits light while in its native state, which significantly reduces the probability of artifacts. Moreover, since the intensity of cell luminescence depends both on the abundance of luciferase and on the presence of substrates for the luciferase reaction, the luminescence of a bacterium is a kind of multiplexer – information from different input channels can be transmitted through one output channel – about the expression of the luciferase operon, on the one hand, and the state of the cell’s energy metabolism, on the other.

The goal of the work is to assess the degree of variability of individual bacterial cells regarding luminescence intensity at different stages of development of batch culture of bacteria, and to test the simplest possible approach to the mathematical description of this variability.

## Experiment description

When growing on rich media, the luminescence intensity of individual cells of brightly luminous strains of luminescent bacteria Photobacterium leiognathi and Ph. phosporeum reaches 104–105 quanta/s. Such signal can be registered using sensitive photometric equipment. The strains used did not demonstrate the typical quorum effect (Brodl et al., 2018) and an increase in their luminescence was observed from the beginning of culture growth.

Without delving into the details of the experimental setup, which operates in the photon counting mode, and the routine for measuring the distribution of bacterial cells according to luminescence intensity (Bartsev, Shenderov, 1985), let us proceed to the description of the results. It should be noted that all experiments were carried out with bacterial clones (genetically homogeneous populations).

During the registration of distributions, the bacteria were in a medium containing only glucose as an energy substrate, i. e. bacterial growth was stopped and the luciferase abundance during the measurement can be considered unchanged. At least, control experiments showed that over a typical period of time the luminescence intensity of individual bacterial cells did not undergo noticeable changes.

**Fig. 1.**
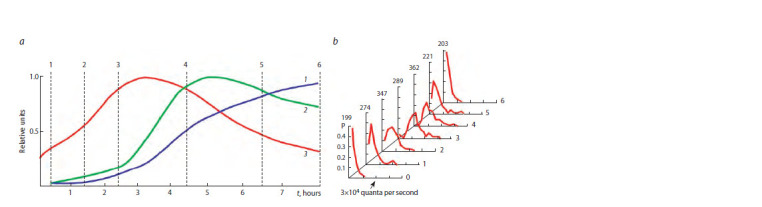
Dynamics of luminescent bacteria culture parameters (a) and cell distributions by luminescence intensity (b). Curves of culture parameters are given in relative units: 1 – optical density; 2 – culture luminescence intensity; 3 – the average intensity of a single cell. The dashed lines indicate sampling times, and their numbers correspond to the numbers of distributions.

A typical view of luminous bacteria distribution at various stages of batch culture growth in a liquid medium is shown in Figure 1.

An immediate question arises regarding the potential mechanism behind the observed variation in the phenotypic trait. The simplest explanation for the observed variability can be suggested immediately – the intensity of the emission is determined by the variability of the bacterial cell volumes. However, direct measurements of cell volume variation in B. subtilis and E. coli showed that the coefficient of variation (CV) of cell volume is ~23 % (van Heerden et al., 2017), while the average CV of bacterial luminescence intensity is ~50 % and can exceed 70 %. Therefore, there is an additional factor that provides a significant variability in cell luminescence

## On possible causes of non-genomic variability

Under normal growth conditions, the luminescence intensity of a bacterial cell is determined by the abundance of luciferase, the enzyme responsible for catalyzing the luminescent reaction, as well as a set of enzymes that supply the necessary substrates for this reaction (Brodl et al., 2018). Proteins involved in bacterial bioluminescence, notably, LuxCDABEG, are encoded by the lux operon and are highly conserved among different bacterial strains. The luxA and luxB genes encode a heterodimeric luciferase; the luxCs, luxDs, and luxE gene products are components of the fatty acid reductase complex; and luxG encodes flavin reductase.

It is natural to assume that in the presence of an energy substrate, as was the case in the experiments performed, the intensity of bacterial luminescence is determined primarily by the expression of the luciferase operon. Other factors, such as the contribution of uneven distribution of protein, mRNA and ribosomes during division, variability in the amount of mRNA due to the small number of molecules, the transition of genes from active to passive state due to reversible binding of a transcription factor, conformation of the DNA molecule that prevents binding RNA polymerases show less variability (Paulsson, 2004; Schwabe, Bruggeman, 2014; Kuwahara et al., 2015; van Heerden et al., 2017; Dessalles et al., 2020) than observed in the experiment. In addition, the resulting cell distributions by protein amount give a distribution close to normal, while asymmetric distributions were observed in the experiment. In addition to this, these distributions demonstrated characteristic dynamics during the development of the enrichment culture, and an adequate model for the formation of distributions of luminescent bacteria by luminescence intensity should, at least qualitatively, reproduce the experimental dynamics.

With a large number of molecules, which is the case for luciferase, fluctuations in its amount between daughter cells are determined by fluctuations in the uneven volumes of daughter cells, which cannot explain the observed CV value. At the same time, it was shown (Taniguchi et al., 2010) that proteins with strong expression have a coefficient of variation of ~30 %, which indicates an “external” factor not associated with fluctuations in a small number of molecules.

## Mathematical model derivation

Without delving into the details of the processes of transcription and translation, let us consider a possible phenomenological stochastic mechanism for generating significant variability in the amount of luciferase in cells. The amount of luciferase in a cell of age τ – z(τ) is the sum of the amount of luciferase received by the cell after division (x) and the amount of luciferase accumulated by age τ – y(τ):

**Formula. 1.**
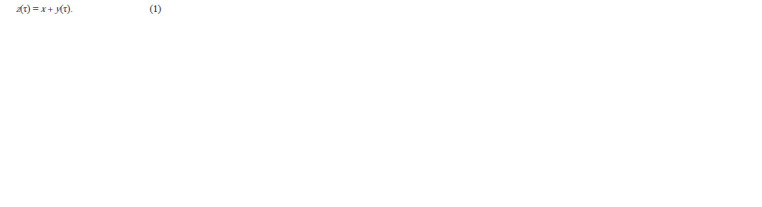
Formula 1

Immediately after division, when τ = 0, the cell contains only the luciferase produced in the previous cell cycle. Let f(x) be the distribution of cells of a narrow age interval according to the amount of luciferase obtained during division, which does not change throughout the entire cell cycle. The form of this distribution is not known and must be obtained by solving the model equation.

Type of cells distribution from a narrow age interval according to the amount of luciferase synthesized and accumulated by age τ – Р( y, τ) can be obtained from the following considerations. For the sake of simplicity, let’s assume that luciferase synthesis begins immediately after cell division, closely associated with the release of DNA from all transcription factors (in our case, the luciferase gene repressor), proceeds at a constant rate, and stops after binding the repressor to the operator.

Let’s assume that τ′ is the moment when the repressor binds to the operator. Then the amount of luciferase synthesized by time τ is described by the following expression:

**Formula. 2.**
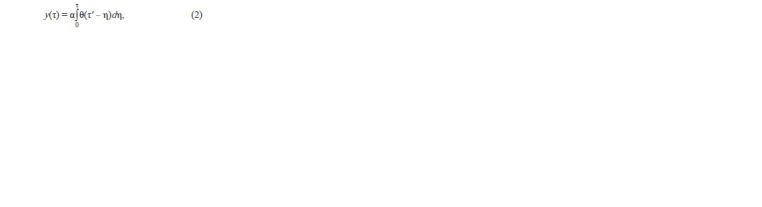
Formula 2

where α is the rate of enzyme synthesis; θ is the Heaviside step function

Since y(τ) is also a function of the random variable τ′, distribution Р( y, τ) is described by the following expression:

**Formula. 3.**
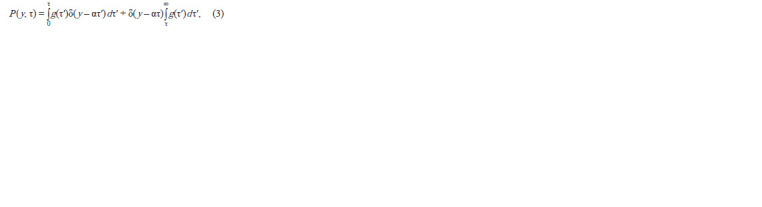
Formula 3

where g(τ′) is the distribution describing the proportion of the cell population in which the binding of the repressor to the operator occurred in the interval [τ′, τ′+dτ′]; δ(x) is the Dirac delta function.

This integral is split into two integrals with integration limits [0, τ) and [τ, ∞), and the cells in which the binding of the repressor to the operator occurred by the age τ (τ′< τ) fall into the first integral, the rest (τ′ ≥ τ) fall into in the second. Let’s do some calculations

**Formula. 4.**
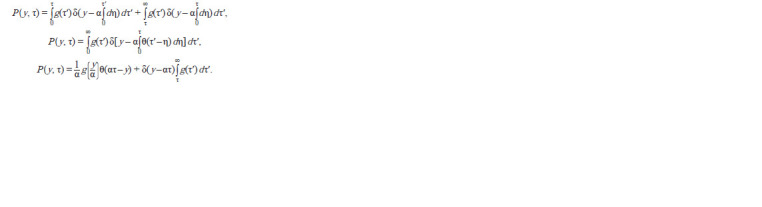
Formula 4

Since the total amount of luciferase in a cell (z(τ)) is the sum of independent random variables x and y, then the distribution of cells in a narrow time interval of age τ by the total amount of luciferase has the following form:

**Formula. 5.**
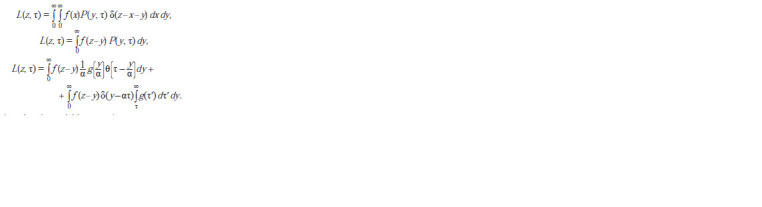
Formula 5

By changing the variables τ′ = y/α we get:

**Formula. 6.**
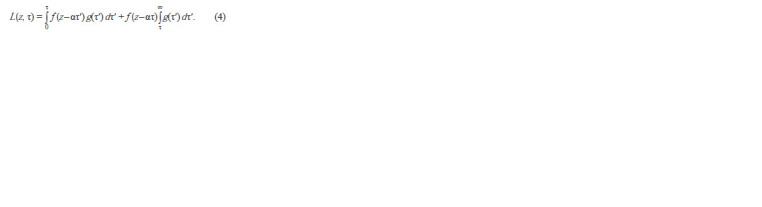
Formula 6

As a result, an expression for the distribution of cells by the amount of luciferase for a narrow age range of age τ was obtained. In order to obtain the equations for the distribution function f (x) and the expression for Φ(z) – the distribution function of the cell population by the amount of luciferase, it is necessary to know the age structure of the population.

The form of cells distribution by age Ψ(τ) is obtained from the equation (Romanovsky et al., 1984):

**Formula. 7.**
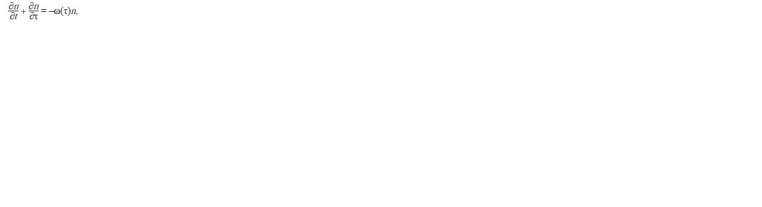
Formula 7

where n(t, τ)dτ is the number of cells of age in the interval [τ, τ + dτ] at the moment t; ω(τ) is the rate of cell loss from a given age interval due to division.

Let us consider the case of a stationary age distribution of bacteria, i. e. n(t, τ)/N(t), is fixed, but the total number of cells N(t) increases. In the case of a stationary distribution, the specific growth rate of cells number in a given age interval is equal to the specific population growth rate:

**Formula. 8.**
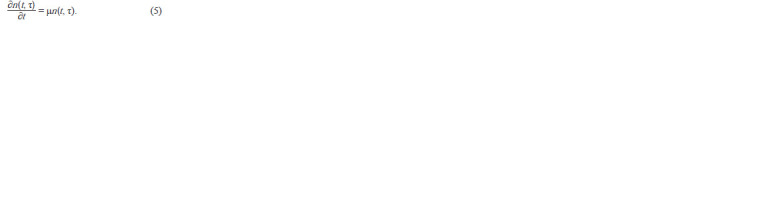
Formula 8

Dividing this equation by N(t) we get the equation for frequencies:

**Formula. 9.**
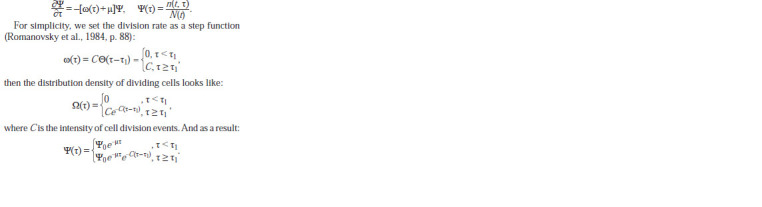
Formula 9

It remains to determine the form of the function g(τ). Assumptions about the constant amount of the repressor in the cell and the irreversibility of its binding to the operator allow us to represent the distribution of cells over the time that elapsed from replication (division) to the moment of binding the repressor to the operator in the form of an exponential distribution:

**Formula. 10.**
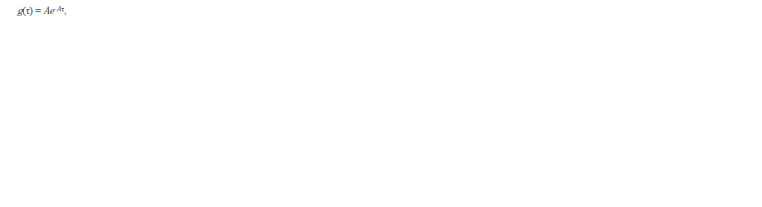
Formula 10

where А is the intensity of events.

As a result of all substitutions, we obtain a model for the distribution of luciferase over the cells of the bacterial culture:

**Formula. 11.**
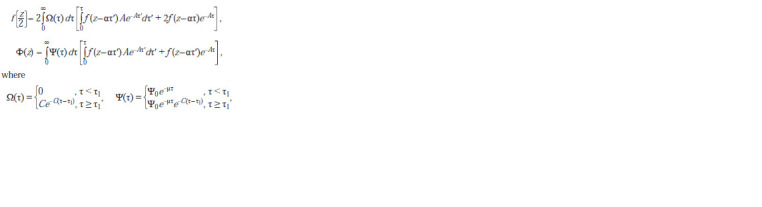
Formula 11

and where f (z) is the density of distribution of cells from a narrow age interval according to the amount of luciferase obtained during division; Ф(z) is the density of cell distribution according to the intracellular amount of luciferase; Ψ(τ) is distribution density of culture cells by age; Ω(τ) is distribution density of dividing cells; A is the intensity of binding the repressor to the operator; α is the rate of luciferase synthesis; C is the intensity of cell division events; τ1 is the minimum age of the beginning of cell division τ.

## Computer simulation

If the resulting equations cannot be solved analytically, then successive approximations are used. But first the values of the model parameters need to be chosen. Note that if the intensity of the repressor binding the activator (parameter A) is equal to zero, then constitutive protein synthesis throughout the entire cell cycle takes place. It is natural to compare this synthesis with the growth of cell volume

That is, the parameters C and τ1 can be determined from other independent distributions (van Heerden et al., 2017), assuming that the coefficients of variation of distributions by volume in luminescent bacteria and other gram-negative bacteria are close. The coefficient of variation of the model distri- bution is close to the value of 24 % at С = 4 and τ1 = 3/4 τ0, where τ0 is the average generation time in the population. These values were used for further simulation. When modeling the dynamics of light intensity distributions during population growth, at the next iteration step the value of the specific growth rate μ was substituted from population growth simulation describing the growth of a real culture

Thus, as a result, there are only two adjustable parameters, or rather, one and a half – the parameter α (the rate of luciferase synthesis) is, in fact, a scale factor. It shows the relative value of the luminescence intensity, mediated in the experiment by the quantum efficiency of the luciferase itself, the geometry of the recording system that determines the amount of light from a bacterium that hits the photocathode of the photomultiplier, the quantum yield of the photocathode, and the fraction of single-electron pulses cut off by the discriminator at the PMT output.

So to describe the dynamics of distributions obtained in the experiment, the model has one adjustable parameter, A, the intensity of repressor-operator binding events. The results of calculations for the most suitable value for describing real distributions, which is A = 2, are shown in Figure 2.

**Fig. 2.**
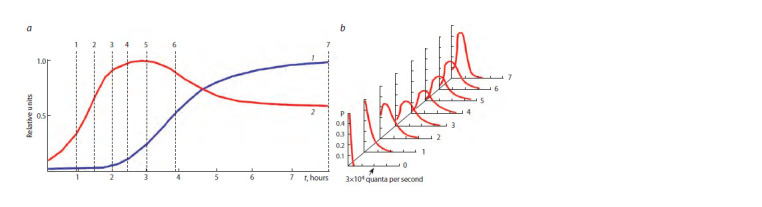
2. Model dynamics of luminescent bacteria culture parameters (a) and cell distributions by luminescence intensity (b). Curves of culture parameters are given in relative units: 1 – biomass; 2 – the average intensity of a single cell emission. The dashed lines indicate the moments of “sampling”, and the numbers correspond to the numbers of the distributions.

When comparing Figures 2 and 1, one can see a quite satisfactory correspondence between them. It is worth noting that this correspondence was obtained with one fitting parameter, which apparently indicates that the proposed model describes something significant in the simulated real system.

It should be noted that luciferase inactivation was not taken into account when deriving the model, which was done to simplify the model; however, it is a common practice (Schwabe, Bruggeman, 2014, p. 306). Palliative inactivation of luciferase can be introduced externally – simply by shifting the distribution points to 0 in proportion to their distance from the origin. In this case, the visual representation of the model would be closer to the experimental data.

However, one property of the model is of interest, which manifested itself in the shift of distributions to 0 at the last stages of population development. By distribution No. 4, the model has almost reached a stationary state and should have remained in it. But since the model takes into account the increase in the duration of the generation time due to the slowdown in culture growth, the established balance between the rate of luciferase synthesis and its distribution between two daughter cells is disturbed.

Since the rate of synthesis of a particular protein is related to the state of basic metabolism, a slowdown in the cell growth rate and accordingly an increase in the generation time leads to a decrease in the rate of luciferase synthesis (decreasing α coefficient). But the intensity of repressor-operator binding events (a physical, energy-independent process) remains the same. However, on the time scale of the cell itself (the unit of measurement is generation time), the rate of luciferase synthesis remained the same, while the intensity of switching events of the luciferase operon increased. Therefore, according to the model, there is a close relationship between the rate of cell growth and the content of luciferase in it, and the higher the rate, the more luciferase is synthesized per cell cycle and vice versa.

The proposed model based on switching off the operon some time after the birth corresponds to the results on the dependence of fluorescent protein expression on cell age (van Heerden et al., 2017, Fig. 4, B, C). It should be noted that the imposition of the age distribution on the expression level curve (Fig. 4, C) was not done entirely correctly by the authors – they have expression even at negative ages (beyond the left border of the age distribution). When bringing the expression level to the age distribution, it would be even more clearly visible, as can be judged by the saturation of the blue area in Fig. 4, B, that the expression level is maximum immediately after the birth of the cell and then decreases with age, which corresponds to the proposed model.

## Conclusion

In conclusion, it can be noted that the proposed phenomenological model with a minimum number of adjustable parameters (1.5) satisfactorily describes a rather complex process that takes place during the growth of a bacterial culture. This may be an indication that the structure of the model describes some essential processes of the real system. Since in the process of division all cells go through the stage of release of all regulatory molecules from the DNA molecule, the resulting distributions can be realized not only in relation to luciferase, but also to other proteins of constitutive (and not only) synthesis

## Conflict of interest

The authors declare no conflict of interest.
